# Changes in Systemic Inflammatory Marker Levels Following Percutaneous Revascularisation of Lower Extremity Arteries

**DOI:** 10.3390/ijms27052404

**Published:** 2026-03-05

**Authors:** Maja Glogovšek, Ula Dobovičnik, Vinko Boc, Anja Boc, Mojca Božič Mijovski, Pavel Poredoš, Kevin Pelicon

**Affiliations:** 1 Department of Vascular Diseases, University Medical Centre Ljubljana, 1000 Ljubljana, Slovenia; 2 Faculty of Medicine, University of Ljubljana, 1000 Ljubljana, Slovenia; 3 Faculty of Pharmacy, University of Ljubljana, 1000 Ljubljana, Slovenia

**Keywords:** peripheral arterial disease, biomarkers, inflammation, endovascular revascularisation, intermittent claudication, atherosclerosis

## Abstract

Peripheral arterial disease (PAD) is a manifestation of systemic atherosclerosis in which inflammation plays a central pathogenic role. Endovascular revascularisation may transiently amplify inflammation due to vascular injury, but successful restoration of perfusion could reduce inflammatory burden over time. This prospective, observational, single-centre pilot study aimed to characterise the temporal dynamics of inflammatory biomarkers during the first three months following endovascular revascularisation of the lower limbs. Consecutive patients with PAD who underwent successful percutaneous femoropopliteal revascularisation at the Department of Vascular Diseases, University Medical Centre Ljubljana, Slovenia, between January 2022 and January 2024 were enrolled. Venous blood was obtained one hour before the procedure, one day afterwards, and again approximately three months later. Concentrations of high-sensitivity C-reactive protein (hsCRP), interleukins (IL-6, IL-8, IL-10), and tumour necrosis factor-alpha (TNFα) were measured. Temporal changes in biomarker levels were analysed using Friedman and Wilcoxon signed-rank tests where appropriate. Clinical outcomes were evaluated at three months and one year post-procedure and were further verified through patient telephone interviews. The observed outcomes were worsening of PAD symptoms, newly diagnosed angina pectoris, myocardial infarction, stroke, transient ischaemic attack (TIA), or death. Twenty-eight patients (median age 69 years) completed all blood samplings. IL-6 concentrations increased significantly one day after revascularisation and decreased below preprocedural levels at three months, with significant differences observed across all time points (*p* < 0.001). IL-10 and TNFα decreased significantly between the postprocedural and three-month measurements (*p* = 0.012 and *p* = 0.016, respectively), but not below preprocedural levels. No significant changes were observed in hsCRP or IL-8. Over a median follow-up of 732 days, 9 patients experienced worsening PAD symptoms in the treated limb, 2 developed new-onset PAD symptoms in the contralateral limb, and 1 was newly diagnosed with angina pectoris. No myocardial infarction, stroke, TIA, or death occurred. To conclude, endovascular femoropopliteal revascularisation induces distinct short-term inflammatory responses, with IL-6 showing the most pronounced peri-procedural dynamics. The observed reductions in some inflammatory biomarker levels at three months suggest that restored limb perfusion may modulate systemic inflammation. Larger studies are warranted to clarify the prognostic relevance of these biomarkers.

## 1. Introduction

Lower-extremity peripheral arterial disease (PAD) is one of the most common clinical manifestations of atherosclerosis [1], which is well-recognised as a chronic, inflammation-driven disease [2,3]. Due to the systemic nature of this pathological process, patients with PAD frequently exhibit concomitant atherosclerotic involvement in other vascular territories, particularly the coronary and carotid arteries [4]. Consequently, their risk of stroke or myocardial infarction is at least comparable to that observed in patients with established coronary artery disease, and they experience substantially elevated rates of both all-cause and cardiovascular mortality [5].

Patients with PAD have elevated baseline levels of inflammatory markers compared with healthy individuals [6,7]. Several of these biomarkers are directly linked to the development, progression, and clinical severity of PAD [6]. Endovascular revascularisation procedures performed to restore blood flow in advanced PAD can modulate systemic inflammation, thereby altering levels of inflammatory biomarkers. A vessel wall injury due to catheter-balloon dilatation or stent implantation can cause an acute rise in circulating inflammatory biomarkers, which, together with platelet and coagulation activation, are considered early indicators of potential restenosis [8]. In the mid- to long-term, however, restored perfusion after successful limb revascularisation can result in a reduction in circulating inflammatory biomarkers [9].

Identifying inflammatory biomarkers that sensitively reflect the impact of endovascular revascularisation on systemic inflammation would enable a better understanding of how changes in inflammatory status influence the incidence of major adverse limb events (MALE) and major adverse cardiovascular events (MACE). Therefore, the present study aimed to investigate the temporal dynamics of circulating inflammatory biomarkers during the first three months following endovascular intervention. We focused specifically on high-sensitivity C-reactive protein (hsCRP), tumour-necrosis factor-alpha (TNFα), and interleukins 6 (IL-6), 8 (IL-8), and 10 (IL-10), which are among the most widely studied markers of systemic inflammation.

## 2. Results

### 2.1. Demographic and Procedural Data

Among the 31 eligible patients initially enrolled in the study, 3 did not complete all three scheduled blood withdrawals and were therefore excluded from further analysis. Table 1 summarises the baseline characteristics of the remaining 28 participants, aged 50–79 years.

Table 2 summarises the characteristics of the treated lesions and the details of the endovascular interventions performed. In one patient, the procedure was complicated by the formation of a femoral pseudoaneurysm, which was managed conservatively.

### 2.2. Laboratory Outcomes

The concentrations of systemic inflammatory biomarkers measured at the three time points, along with *p*-values for overall comparisons across all blood samplings, are shown in Table 3. While all participants were sampled one hour before and on the day after the procedure, the third sampling was performed 43–135 days post-procedure (median 81 days; IQR 63–99 days).

**Figure 1:**
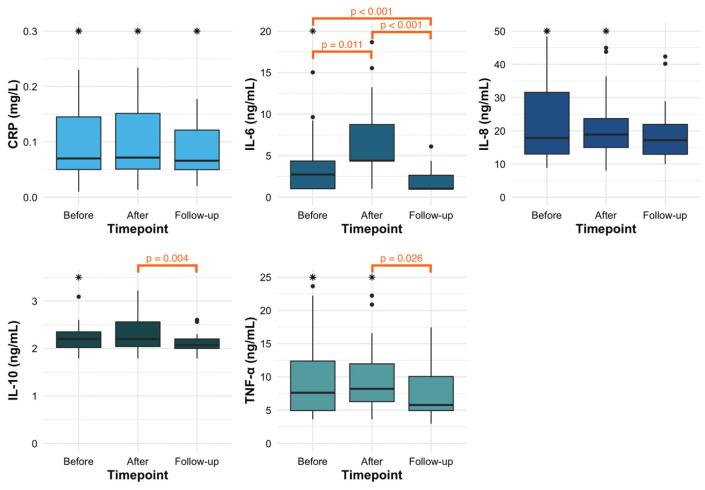
Concentrations of high-sensitivity C-reactive protein (CRP), interleukin 6 (IL-6), interleukin 8 (IL-8), interleukin 10 (IL-10), and tumour necrosis factor-alpha (TNFα) at one hour before the procedure (Before), one day after the procedure (After), and three months after the procedure (Follow-up). Boxplots show the median and interquartile range (IQR); whiskers indicate the range excluding outliers, while black dots represent outliers (values outside 1.5 × IQR). Asterisks signify outliers that fall outside the y-axis. Only significant *p*-values are presented.

Pairwise comparisons between sampling time points were performed for biomarkers that showed statistically significant differences in the overall analysis and are presented in Figure 1. For IL-6, the differences were most pronounced, with statistically significant changes observed between all three time points. In contrast, for IL-10 and TNFα, significant differences were found only between the measurements taken one day and three months after the procedure.

### 2.3. Long-Term Outcomes

Over a mean follow-up of 732 days (range 214 to 1105), 12 patients experienced one of the observed adverse outcomes. In 9 patients, claudication in the treated lower limb worsened, and 4 of them underwent repeat revascularisation. An additional 2 patients developed new-onset PAD symptoms in the contralateral limb, and 1 patient was newly diagnosed with angina pectoris. No myocardial infarction, stroke, TIA, or death was recorded.

## 3. Discussion

This study aimed to investigate the effects of endovascular revascularisation of the femoropopliteal arterial segment on the temporal dynamics of inflammatory biomarkers hsCRP, IL-6, IL-8, IL-10, and TNFα in the bloodstream. Among these, IL-6 was most affected by the procedure, with its levels increasing significantly one day after the intervention and subsequently decreasing significantly during follow-up, both compared with the preprocedural and postprocedural concentrations. The intervention also affected IL-10 and TNFα levels, which were significantly lower three months after the procedure compared to one day after the procedure, although not significantly different from the preprocedural baseline level, suggesting only resolution of the acute inflammatory response rather than a long-term systemic anti-inflammatory effect of revasculariation.

According to our results, IL-6 appears to be a particularly sensitive marker of inflammatory response to endovascular intervention. This pro-inflammatory cytokine is a central mediator linking the acute-phase response to chronic inflammation, stimulating the synthesis of acute-phase proteins and regulating immune cell differentiation [12,13]. Within the cardiovascular system, IL-6 contributes to endothelial dysfunction, monocyte activation, and adhesion molecule expression, while stimulating CRP production, all of which promote atherogenesis and postprocedural restenosis [14,15]. Its significance has also been established in PAD, where elevated IL-6 concentrations have been detected even in asymptomatic patients [16], and are predictive of future cardiovascular events and mortality [17]. Consistent with our findings, previous studies have demonstrated transient post-revascularisation increases in IL-6. Araújo et al. reported elevated IL-6 levels shortly after revascularisation, followed by a decline at 6 months [18], while Takamura et al. observed a progressive rise within the first 24 h post-procedure, with levels remaining elevated at 36 h [19]. Although Araújo et al. found no significant relationship between IL-6 levels and restenosis after peripheral artery revascularisation [18], studies in patients undergoing coronary angioplasty have demonstrated a positive association [20]. Although the influence of IL-6 on cardiovascular events and mortality is well established [17], we did not identify any studies that specifically investigated the impact of post-procedure IL-6 reduction on major cardiovascular events. In our cohort, 11 patients experienced progression of PAD in either the treated or contralateral limb, and 1 patient was first diagnosed with ischaemic cardiac disease, while no deaths occurred. However, the number of events was too small and the follow-up too short to permit meaningful statistical analysis.

Among the acute-phase proteins stimulated by IL-6 is CRP, which actively contributes to all stages of atherosclerosis by promoting vascular inflammation, lipid accumulation, thrombosis, modulation of leukocyte recruitment, endothelial activation, and platelet aggregation [21]. In patients with PAD, higher CRP concentrations are associated with disease severity and progression [22], and can help predict future arterial and cardiovascular events [23]. While we did not observe significant changes in CRP levels following revascularisation, some of the previous studies have demonstrated its acute postprocedural increase [24,25]. The discrepancy between our IL-6 and hsCRP findings may partly reflect a type II error due to limited statistical power. However, biological factors should also be considered. While IL-6 increases within hours after an inflammatory stimulus [26,27], CRP demonstrates slower kinetics, typically peaking after 24–72 h [28]. Consequently, the timing of our second sampling may not have fully captured a transient CRP peak, potentially contributing to the absence of a statistically significant change.

In contrast to earlier reports describing a reduction in IL-8 concentrations following revascularisation [18,29], our study did not demonstrate a significant change in IL-8 levels. Direct comparison between studies is challenging due to differences in patient populations, sampling time points, and overall study design. While Araújo and colleagues included both patients with intermittent claudication and those with CLTI (with more than 50% presenting with ulcers), Poredoš et al., as well as our cohort, included only patients with limiting claudication. These patients are generally associated with lower systemic inflammation and may therefore exhibit a more attenuated inflammatory response to revascularisation. Moreover, Araújo and colleagues assessed biomarker levels after 6 months, whereas our study used a shorter follow-up interval, possibly influencing the detection of dynamic inflammatory changes. Importantly, all available studies in this field have relatively small sample sizes, limiting statistical power and increasing the risk of both type I and type II errors. Thus, the reported discrepancies may partly reflect limited sample sizes rather than true biological differences. Additionally, subtle variations in procedural techniques, completeness of revascularisation, and peri-procedural management may further contribute to heterogeneity in inflammatory responses. Within the cardiovascular system, IL-8 contributes to atherosclerosis by enhancing leukocyte adhesion, smooth muscle cell proliferation, and angiogenesis, linking inflammation to plaque progression and instability [30]. Previous studies have reported that elevated IL-8 levels predict major adverse limb events in patients with chronic limb-threatening ischaemia, highlighting its potential role as a biomarker of inflammatory disease progression and poor clinical outcome [31].

TNFα is another pro-inflammatory cytokine, inducing endothelial dysfunction and leukocyte recruitment, and promoting vascular smooth muscle cell proliferation and matrix remodelling [32]. TNFα levels are elevated in PAD patients [16], and have been associated with reduced maximal walking time in those with intermittent claudication [33]. Moreover, Li et al. identified TNFα as a biomarker predictive of MACE over a 2-year follow-up in patients with PAD [34]. In our study, revascularisation did not induce an acute change in TNFα levels, and a significant decrease three months after the procedure was observed only in comparison with the 24 h postprocedural levels, but not when compared to the preprocedural baseline.

IL-10 is a key anti-inflammatory cytokine that counterbalances the pro-inflammatory response triggered by vascular injury. Within the cardiovascular system, IL-10 stabilises atherosclerotic plaques, reduces oxidative stress, and attenuates endothelial dysfunction. While its predictive value has not been confirmed in individuals without clinical heart disease [35], high plasma levels of IL-10 were associated with a better prognosis in patients with acute coronary syndrome [36]. Studies evaluating IL-10 levels in patients with PAD are limited. In our study, we did not confirm a significant increase in IL-10 following revascularisation in comparison to preprocedural levels. This could suggest that the anti-inflammatory response mediated by IL-10 is unaffected by revascularisation, or that IL-10 levels increase more gradually after the intervention.

This study has several limitations. Being a pilot study, it has a small sample size, which reduces statistical power and limits the generalisability of the findings. Although the use of repeated measurements within the same individuals over time partially mitigated inter-individual variability, the limited sample size constrained our ability to fully account for and adjust for the potential influence of atherosclerotic risk factors and comorbidities, as well as prescribed medication, including immunomodulatory and antithrombotic drugs. The low number of clinical events also precluded any meaningful statistical outcome-based analysis. Moreover, the cohort was recruited from a single-centre population in Slovenia, which is predominantly white, and approximately two-thirds of participants were male. These demographic characteristics restrict external validity and highlight the need to confirm our findings in larger, more diverse cohorts.

## 4. Materials and Methods

This prospective, observational, single-centre pilot study was conducted in the Catheterisation Laboratory of the Department of Vascular Diseases at the University Medical Centre Ljubljana, Slovenia. Between January 2022 and January 2024, consecutive PAD patients who underwent successful percutaneous revascularisation of the native femoropopliteal segment for lifestyle-limiting intermittent claudication were included. Exclusion criteria comprised the presence of symptomatic PAD in the contralateral limb, chronic limb-threatening ischaemia (CLTI), any systemic inflammatory disease, or inability to ambulate independently.

During the intervention, a pre-revascularisation angiogram was recorded to assess the complexity of the lesion for the Trans-Atlantic Inter-Society Consensus Document on Management of Peripheral Arterial Disease II (TASC II) classification [11]. Lesions were primarily treated with balloon angioplasty. Bailout stenting was performed only in cases of significant residual stenosis, early elastic recoil, or flow-limiting arterial wall dissection. Drug-coated balloons were used at the operator’s discretion.

In all participants, the ankle-brachial index (ABI) was measured before revascularisation and again one day after the procedure. Risk factors for atherosclerosis and associated cardiovascular illnesses were documented. In patients with suboptimal treatment regimes, the pharmacological therapy was optimised according to current guideline recommendations [37]. After the procedure, patients were prescribed antithrombotic therapy according to protocol [38].

All participants were re-evaluated at three months and one year post-revascularisation. Follow-up assessments included clinical examinations, patient-reported symptoms, and ABI measurement. Long-term outcomes were verified through a review of the latest electronic medical records and structured telephone interviews conducted in February 2025. During these interviews, patients provided information on their current health status, functional capacity, walking distance, adherence to prescribed therapy, and any recent hospitalisations or new diagnoses not yet recorded in the hospital information system. The observed outcomes were worsening of PAD symptoms on the treated or contralateral limb, newly diagnosed angina pectoris, myocardial infarction, stroke, transient ischaemic attack, or death. The study was approved by the National Medical Ethics Committee (number 0120-607/2021/4) and conducted in accordance with the principles of the Declaration of Helsinki. Before study enrolment, all participants were informed of the study procedures and provided written informed consent.

### 4.1. Laboratory Investigations

Three venous blood samples were collected from each participant via the median cubital vein into vacuum tubes containing a clot activator: the first sample was taken 1 h before the revascularisation procedure, the second 1 day after the procedure, and the third was planned for a follow-up visit 3 months after the procedure. Serum was obtained by centrifugation (2000× *g* for 15 min at 20 °C) within one hour of blood collection, aliquoted into cryovials, frozen, and stored at ≤−70 °C until analysis. Serum samples were thawed just prior to analysis at 37 °C for 5–10 min and vortexed. One serum aliquot was used to measure the concentration of hsCRP (1-plex, sensitivity 1.4 ng/L, intra-assay CV < 17%). Another serum aliquot was used to measure concentrations of IL-6, IL-8, IL-10, and TNFα (4-plex, with the sensitivities of 1.1, 2.0, 0.3, and 1.5 ng/L, respectively and intra-assay CVs 2.2, 3.2, 3.6 and 3.0%, respectively). All measurements were performed in a single run using xMAP© technology with magnetic beads coupled to specific antibodies (all from R&D Systems, Minneapolis, MN, USA) on a MagPix instrument (Luminex Corporation, Austin, TX, USA).

### 4.2. Statistical Analysis

Statistical analyses were performed using GraphPad Prism version 10.5.0 for Windows (GraphPad Software, San Diego, CA, USA). The normality of data distribution was assessed using the Shapiro–Wilk test. As the distribution of all systemic inflammatory biomarkers was skewed, non-parametric statistical tests were applied. Concentrations are presented as medians with interquartile ranges (IQR). Differences across the three time points were analysed using the Friedman test, followed by pairwise comparison without correction using the Wilcoxon signed-rank test for variables showing significant results in the Friedman test. Box plots of biomarker values at each of the three time points (Figure 1) were plotted using RStudio version 2025.05.0+496 and the R programming language version 4.5.0 (R Foundation for Statistical Computing, Vienna, Austria) [39].

## 5. Conclusions

Endovascular femoropopliteal revascularisation in patients with intermittent claudication was associated with distinct alterations in inflammatory marker levels, characterised by an acute peri-procedural rise in IL-6 and a subsequent decline in IL-6, IL-10, and TNFα at three months. These findings suggest that successful restoration of blood flow may not only enhance functional capacity but also modulate systemic inflammation, thereby potentially slowing the progression of both local and systemic atherosclerosis. The observed changes in biomarker levels should, however, be interpreted cautiously given the limited sample size and exploratory nature of this study. Based on our results, IL-6 appears to be a particularly sensitive circulating marker of the inflammatory response to endovascular intervention and may serve as a useful indicator for exploring the relationship between procedure-induced inflammation, arterial patency, and the evolution of atherosclerotic disease in other vascular territories. More research is needed to confirm the observed temporal changes in biomarkers under consideration and to determine their potential role in predicting outcomes of lower-extremity PAD.

## Figures and Tables

**Table 1:** Baseline characteristics of the 28 study participants.

Patient Demographics	
Age, years	69 (60–73)
Male sex	18 (64.3)
**Cardiovascular risk factors and associated illnesses**	
Arterial hypertension	25 (89.3)
Dyslipidaemia	28 (100)
Diabetes mellitus	12 (42.9)
Smoking	23 (82.1)
active	12 (42.9)
former	11 (39.3)
Chronic kidney disease (eGFR < 60 mL/min/1.73 m^2^)	5 (17.6)
Ischaemic heart disease	7 (25.0)
Prior stroke or TIA	3 (10.7)
Previous peripheral artery revascularisation	14 (50.0)
**Prescribed medication**	
Aspirin only	11 (39.3)
Aspirin and low-dose rivaroxaban	13 (46.4)
Aspirin and clopidogrel	1 (3.6)
Therapeutic doses of anticoagulants (warfarin or rivaroxaban) *	3 (10.7)
Statin only	22 (78.6)
Statin and ezetimibe	5 (17.9)
PCSK-9 inhibitor and ezetimibe	1 (3.6)
Antihypertensive medication	24 (85.7)

Data are presented as the number and proportion of subjects (N (%)), and as the median and interquartile range (IQR) for age. Due to rounding, totals may differ from 100%. * Patients were prescribed therapeutic doses of anticoagulants due to other indications, such as atrial fibrillation or prior thromboembolic events. eGFR: estimated glomerular filtration rate, calculated by the MDRD formula [10]; TIA: transitory ischaemic attack; PCSK-9: proprotein convertase subtilisin/kexin type 9.

**Table 2:** Characteristics of the treated lesions and of the endovascular interventions.

Lesion Characteristics	
Stenosis/occlusion	16 (57.1)/12 (42.9)
TASC II A	4 (14.3)
TASC II B	8 (28.6)
TASC II C	14 (50.0)
TASC II D	2 (7.1)
**Procedure characteristics**	
Stent placement	3 (10.7)
Use of a paclitaxel-eluting balloon	8 (28.6)

Data are presented as the number and proportion (N (%)). TASC II: Trans-Atlantic Inter-Society Consensus II [11].

**Table 3:** Concentrations of the observed inflammatory biomarkers at one hour before the procedure (1st blood sample), one day after the procedure (2nd blood sample), and approximately three months after the procedure (3rd blood sample).

	1st Blood Sample	2nd Blood Sample	3rd Blood Sample	*p*-Value
hsCRP (mg/L)	0.06 (0.05–0.15)	0.07 (0.04–0.15)	0.07 (0.05–0.45)	NS
IL-6 (ng/L)	2.7 (1.0–4.4)	4.4 (4.4–10.5)	1.0 (1.0–2.6)	<0.001
IL-8 (ng/L)	17.4 (13.0–32.3)	19.2 (15.2–24.8)	17.2 (12.8–22.3)	NS
IL-10 (ng/L)	2.2 (2.0–2.3)	2.2 (2.0–2.6)	2.0 (2.0–2.2)	0.012
TNFα (ng/L)	7.5 (4.9–12.4)	8.3 (7.0–13.0)	5.8 (4.9–10.5)	0.016

Data are presented as the medians (interquartile ranges). The *p*-value refers to the comparison across all three samplings for each inflammatory biomarker. hsCRP: high-sensitivity C-reactive protein; IL: interleukin; NS: not significant; TNFα: tumour necrosis factor alpha.

## Data Availability

The underlying data used for this article can be shared upon reasonable request.

## Abbreviations

The following abbreviations are used in this manuscript:

ABI	Ankle-brachial index
CLTI	Chronic limb-threatening ischaemia
CRP	C-reactive protein
eGFR	Estimated glomerular filtration rate
PAD	Peripheral arterial disease
IL	Interleukin
IQR	Interquartile range
MACE	Major adverse cardiovascular event
MALE	Major adverse limb event
PCKS-9	Proprotein convertase subtilisin/kexin type 9
TASC II	Trans-Atlantic Inter-Society Consensus II
TIA	Transitory ischaemic attack
TNFα	Tumour necrosis factor-alpha
